# Chondrosarcoma of the Sternum: Surgical Challenges, Chest Wall Reconstruction, and Postoperative Management

**DOI:** 10.7759/cureus.57594

**Published:** 2024-04-04

**Authors:** Nina Trepić, Marko Nemet, Ivan Ergelašev

**Affiliations:** 1 Internal Medicine, Faculty of Medicine, University of Novi Sad, Novi Sad, SRB; 2 Thoracic Surgery, Institute for Pulmonary Diseases of Vojvodina, Sremska Kamenica, SRB; 3 Surgery, Faculty of Medicine, University of Novi Sad, Novi Sad, SRB

**Keywords:** thoracic wall, sternum, reconstructive surgical procedures, postoperative complications, chondrosarcoma

## Abstract

Although rare, primary chondrosarcoma is the most frequent malignant tumor of the sternum. It commonly manifests as a painful, expanding mass arising from the costochondrosternal junction. Since it is resistant to radiotherapy and chemotherapy, surgical resection with reconstruction is the preferred treatment. A 50-year-old male presented with swelling over the left fourth sternocostal joint, gradually increasing in size. Imaging and clinical assessment suggested an infiltrative neoplasm, and surgical resection was indicated. The patient underwent a partial sternectomy, including a resection of the xiphoid process and costal cartilages two to seven and a partial resection of the manubrium. Postoperative pathohistological analysis specified the change as a low-grade chondrosarcoma in the pT1 stage. Chest wall reconstruction involved three pectus bars fixated around the ribs and the placement of a synthetic polypropylene mesh. The patient required postoperative rehospitalization due to partial skin layer wound dehiscence, serous drainage, and fever. Empirical antibiotic therapy was initiated, and the patient underwent a median superior laparotomy with partial omentoplasty of the sternal region, preserving the mesh and pectus bars. A culture analysis revealed methicillin-resistant *Staphylococcus epidermidis*, and postoperative antibiotic therapy was adapted to the antibiogram. Subsequently, all parameters of inflammation decreased, and wound healing followed. A one-year follow-up CT scan showed no disease recurrence. This case highlights the intricate surgical management that contributed to the successful treatment of sternal chondrosarcoma. Sternal wound infection, a severe postoperative complication with a high mortality rate, requires prompt identification, precise revision with culture-directed antibiotics, and effort to preserve the prosthetic material.

## Introduction

Primary tumors affecting the chest wall are a rare occurrence, representing less than 2% of all primary tumors, with about 50-80% of these being malignant [1]. More frequently, malignancies involving the chest wall are found to be secondary metastases from other organs [2]. Among these chest wall tumors, chondrosarcoma stands out as the predominant entity. It is characterized by a distinct clinical presentation featuring an enlarging mass, often observed on the anterior aspect of the chest wall, most frequently in the superior five ribs, and proximity to the costochondral junction [3].

The management of malignant chest wall tumors poses unique challenges, particularly in the case of chondrosarcoma, where effective treatment options, such as chemotherapy and radiotherapy, are limited. The gold standard for diagnosing chondrosarcoma is the CT scan, which provides detailed insights into the distinctive features of this tumor. These imaging studies reveal a well-defined, lobulated soft-tissue mass with a calcified chondroid matrix, facilitating accurate identification and assessment [4]. However, the diagnostic journey extends beyond imaging, as exemplified by our presented case.

The challenges posed by chondrosarcoma, including its poor vascularity and intricate extracellular matrix, making it resistant to chemotherapy and radiation therapy [5], necessitate surgical resection [6]. Tumors affecting this region demand careful consideration due to their potential to significantly influence both physiological and anatomical aspects of the thoracic cavity. The partial or total removal of the sternum, resulting in large defects in the chest wall, presents a significant surgical challenge due to difficulties in reconstruction to preserve stability and adequate lung function [7]. Reconstruction following wide sternectomy in the case of anterior or lateral defects larger than 4-5 cm requires the use of rigid prosthetic materials to achieve chest wall stability [8].

## Case presentation

**Figure 1:**
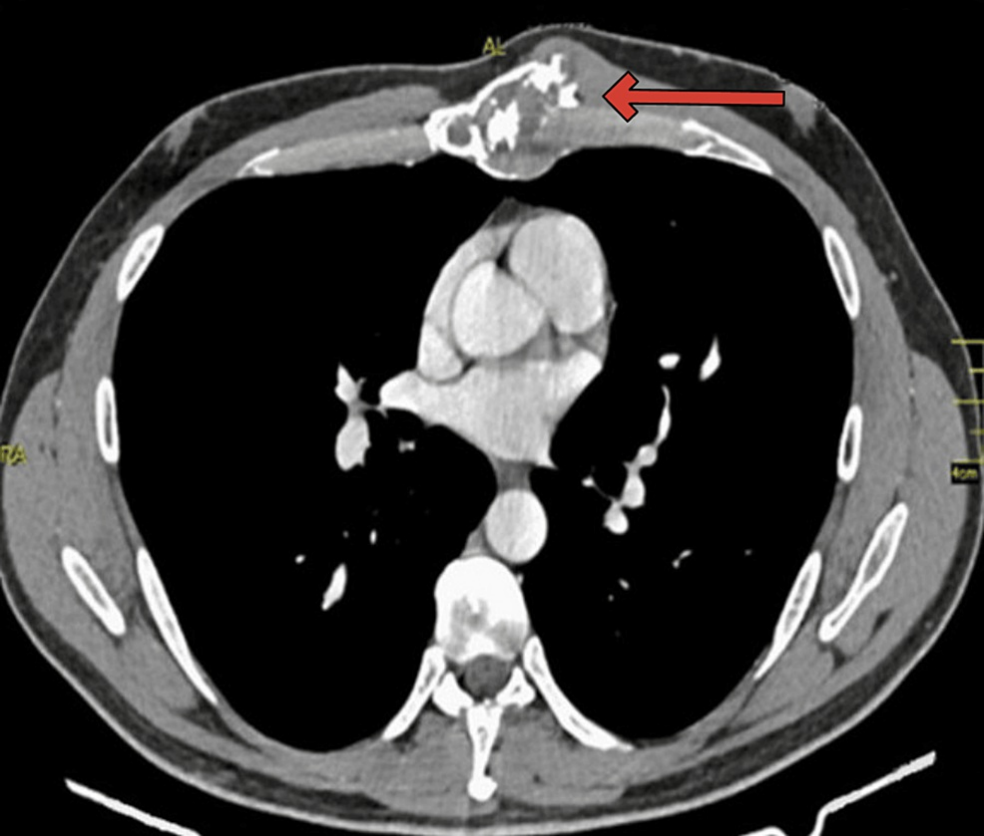
CT scan of the thorax revealing an expansive process in the sternum The chest CT image in a soft tissue window setting shows an expansive process in the sternum with coarse mineralization, indicated by a red arrow.

A 50-year-old male presented with swelling over the left fourth sternocostal joint, gradually increasing in size. A CT scan of the thorax revealed an expansive process in the sternum with coarse mineralization inside the medullary matrix (Figure 1).

**Figure 2:**
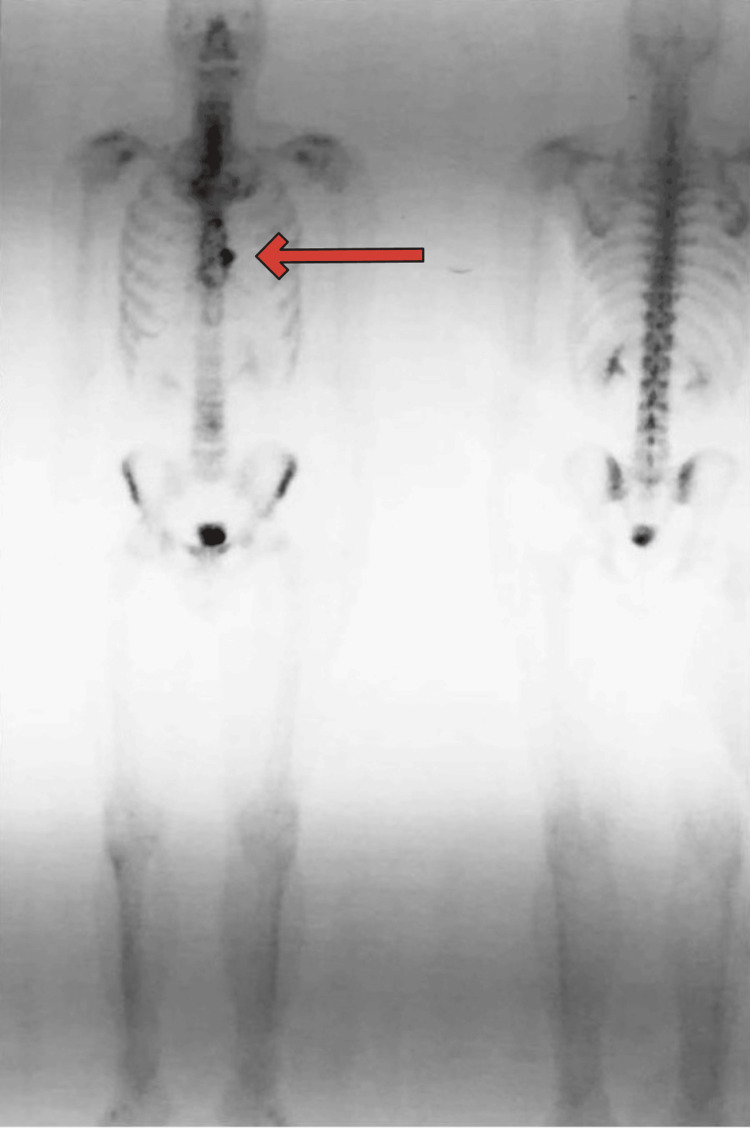
Scintigraphy showing hyperfixation of the radiopharmaceutical in the body of the sternum Bone scintigraphy using Technetium-99m shows pathological hyperfixation in the body of the sternum, as indicated by a red arrow.

Additionally, a tumorous outgrowth anterolaterally on the left side, also calcified, was observed. Subsequent scintigraphy showed pathological hyperfixation of the radiopharmaceutical in the body of the sternum without any evidence of skeletal pathologic accumulation elsewhere (Figure 2).

**Figure 3:**
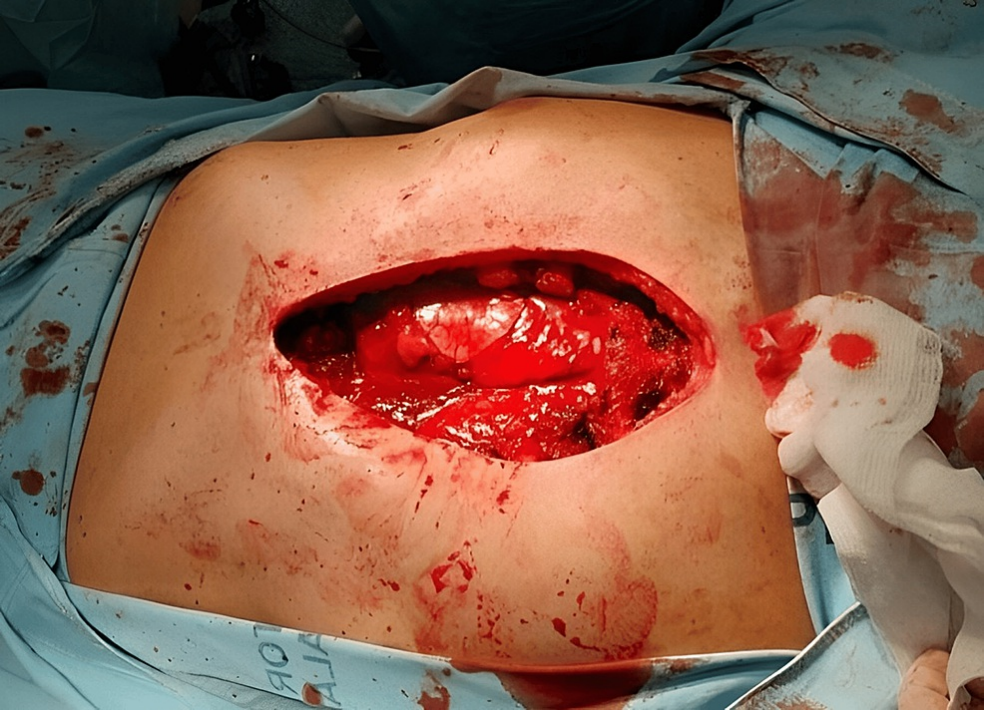
Partial resection of the sternum with costal cartilages two to seven

To eliminate alternative diagnoses, the determination of Bence-Jones proteins was pursued, with negative results. Imaging and clinical assessment suggested an infiltrative neoplasm, prompting surgical intervention. The patient underwent a partial sternectomy, including the resection of the xiphoid process and costal cartilages two to seven on both sides (Figure 3).

**Figure 4:**
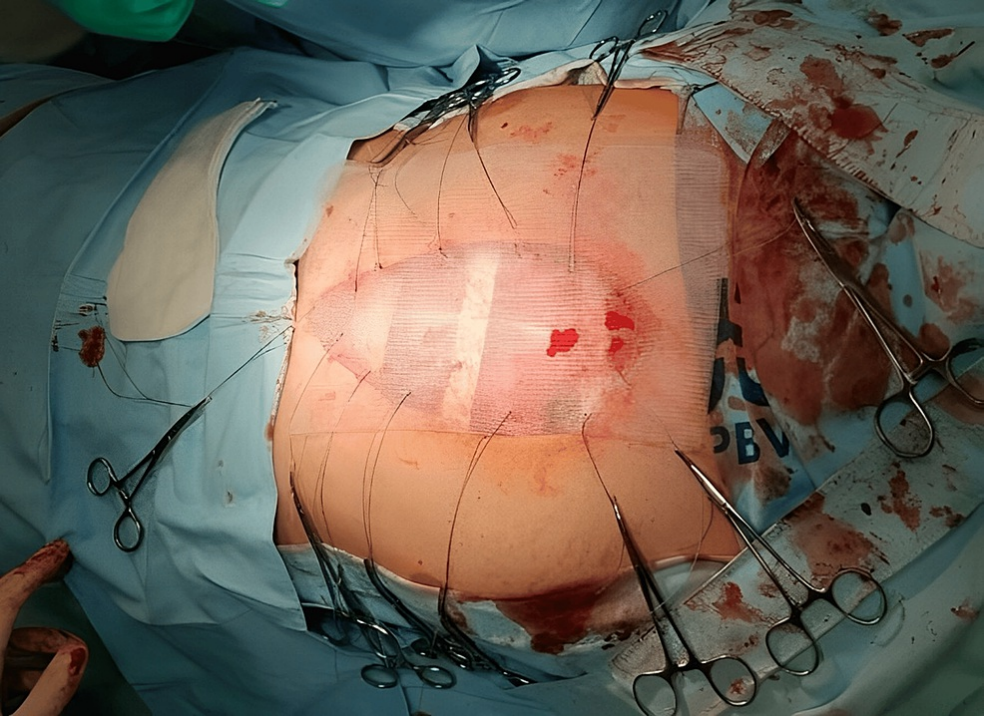
Reconstruction of the chest wall with a polypropylene mesh

**Figure 5:**
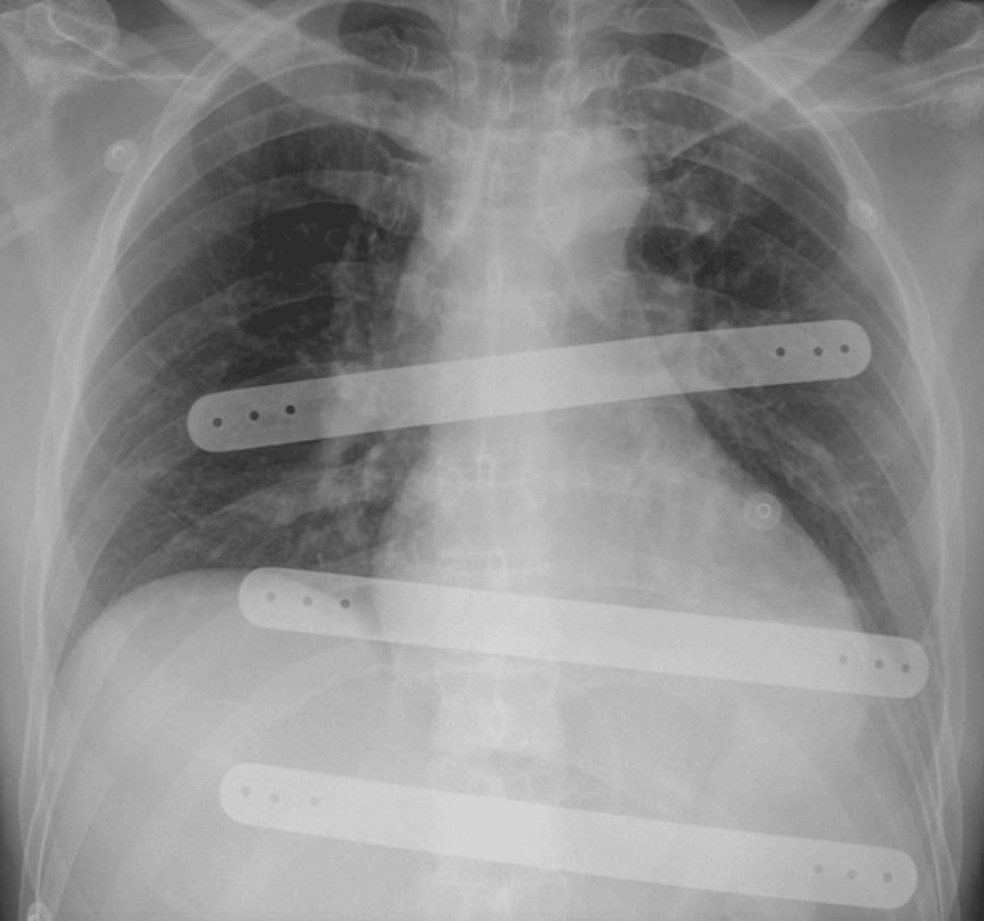
X-ray showing pectus bars used for reconstruction of the chest wall

Due to the presence of tumor tissue at the manubriosternal junction, the manubrium was partially excised, leaving only the attachment of the first rib to stabilize the clavicles. Multiple biopsies were obtained for ex tempore analysis, indicating a soft tissue tumor. The remaining resected tissue underwent definite pathohistological analysis. Chest wall reconstruction involved the placement of three pectus bars fixated around the ribs and a synthetic polypropylene mesh sewn onto the remaining ribs (Figures 4, 5).

**Figure 6:**
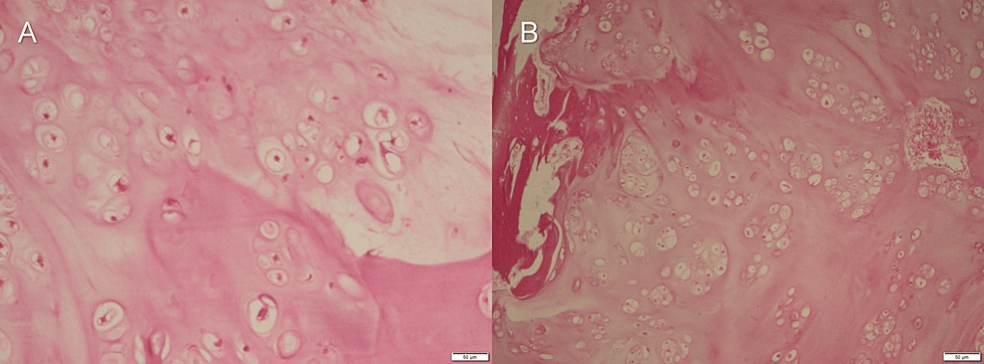
Histopathology images confirming the diagnosis of chondrosarcoma Microscopic examination after decalcification reveals chondrosarcoma: (A) H&E staining, x10; (B) H&E staining, x5

Subsequent definite pathohistological analysis, which included decalcification and staining with H&E, identified the tumor as a low-grade chondrosarcoma in the pT1 stage of the disease (Figure 6).

**Figure 7:**
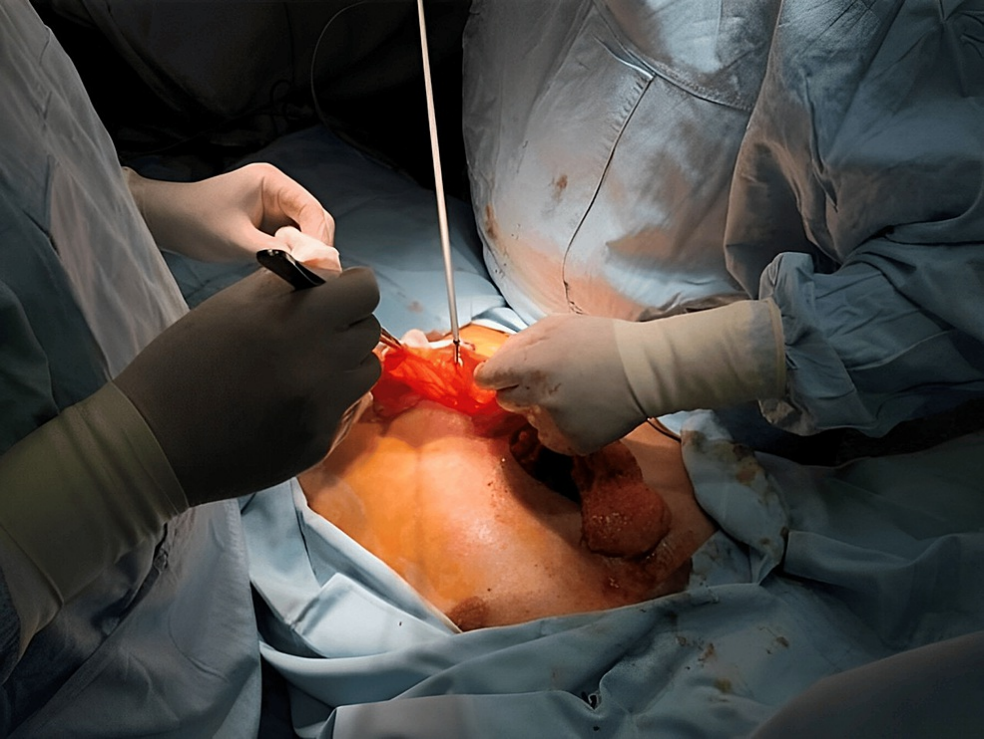
Wound revision via a median superior laparotomy with partial omentoplasty

Furthermore, pathohistological analysis confirmed complete tumor resection (R0). Antibiotics were prescribed according to the protocol of our institution for operative treatment. Cefuroxime 1.5 g was given three times a day for three days, with the first dose given intraoperatively. Following a favorable postoperative course, the patient was discharged on the 10th postoperative day. Five days after discharge, the patient presented to the emergency department and required readmission to the hospital due to serous discharge from the lower part of the incision, partial dehiscence of a skin layer, and fever. Management of these postoperative complications necessitated the initiation of empirical antibiotic therapy with Clindamycin. A second operation for wound revision was performed via a median superior laparotomy with partial omentoplasty, preserving the mesh and pectus bars (Figure 7).

**Figure 8:**
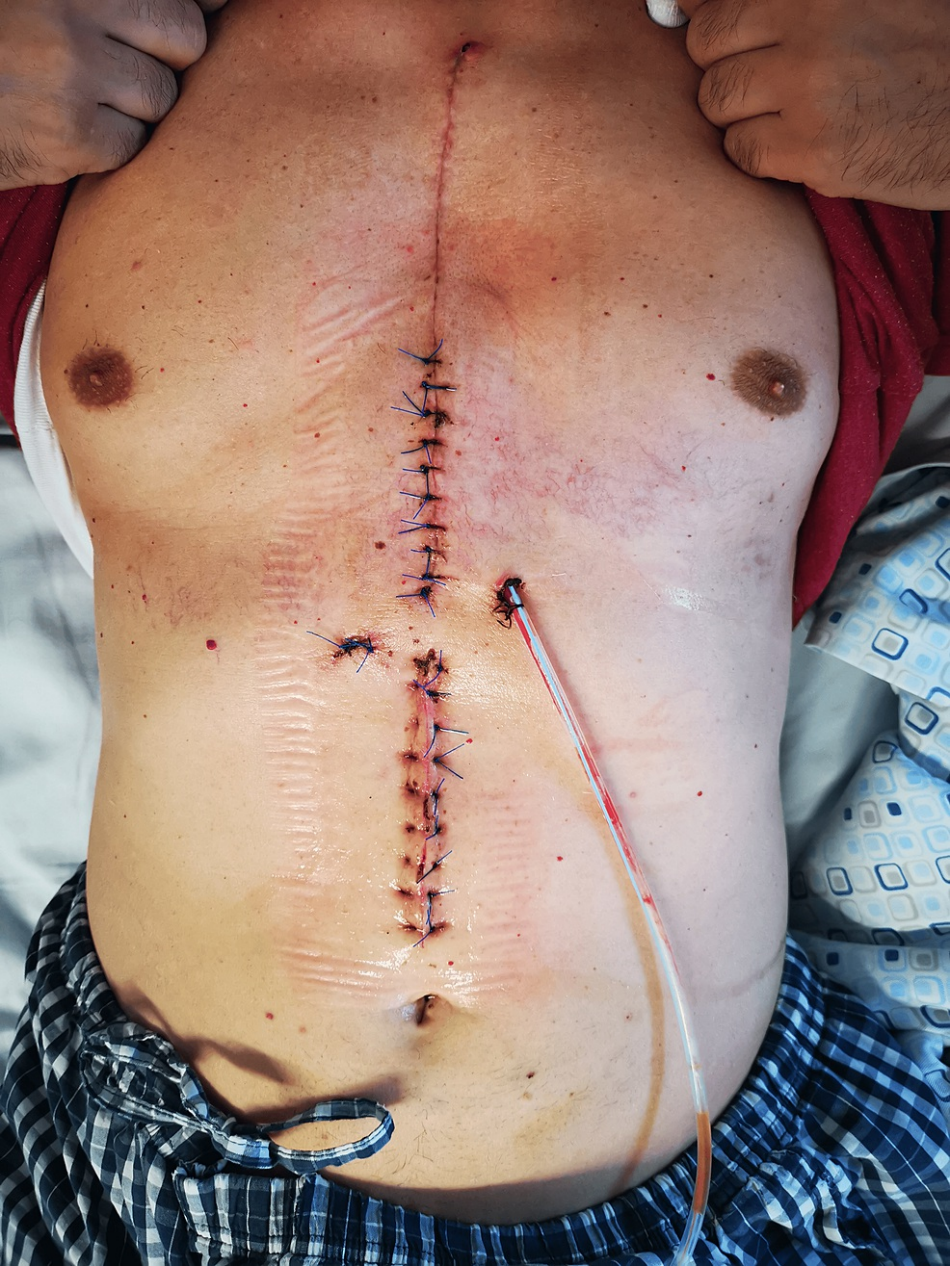
The surgical site after the second discharge

Intraoperatively, there were no signs of infection with the prosthetic material. Culture analysis yielded a positive result for methicillin-resistant *Staphylococcus epidermidis*, leading to an adjustment of antibiotic therapy. Based on the corresponding antibiogram, the patient received 10 days of Linezolid therapy. Subsequently, inflammatory parameters normalized, and the wound continued to heal (Figure 8).

A one-year follow-up CT revealed no disease recurrence. There were no indications for postoperative radiation therapy, with regular follow-ups being recommended.

## Discussion

Primary malignant tumors of the chest wall present challenges when it comes to diagnosis and treatment. Chondrosarcoma, in particular, requires consideration due to its distinctive clinical features and limited treatment options [3].

The clinical presentation of an enlarging mass on the anterior wall of the chest observed in our patient aligns with the typical characteristics of chondrosarcoma, as described in existing literature [3]. A gold standard CT scan revealed distinct morphological characteristics, such as a well-defined, lobulated mass with a calcified chondroid matrix, highlighting the importance of imaging [4]. While imaging plays a role in diagnosis, our case underscores that reaching a diagnosis involves more than radiological findings.

Based on the clinical presentation, a thorough investigation was started to rule out the possibility of multiple myeloma. The assessment of Bence-Jones proteins, taking into account factors like the patient’s age and clinical and radiological findings, played a role in this process. The subsequent negative results for Bence-Jones proteins helped to exclude multiple myeloma as the primary pathology, shifting the diagnostic focus toward chondrosarcoma.

Considering that chondrosarcoma does not respond well to chemotherapy or radiation therapy, the National Comprehensive Cancer Network (NCCN) Guidelines recommend excision as the treatment approach for a complete cure (Table 1).

**Table 1:** Summary of NCCN Clinical Practice Guidelines for the treatment of chondrosarcoma Clinical Practice Guidelines in Oncology – version 2.2024 for Chondrosarcoma by the NCCN [9] NCCN, National Comprehensive Cancer Network

Presentation	Low-grade tumors originating from extracompartmental appendicular areas, grade I tumors located in axial regions, high-grade tumors (grade ll and grade lll), clear cell tumors, and extracompartmental tumors
Primary treatment	Surgical removal with wide excision is recommended for resectable tumors. For tumors that are borderline resectable or unresectable, consider radiotherapy as an alternative option.
Surveillance	Regular physical examinations are recommended. Imaging studies such as X-rays of the primary site and/or cross-sectional imaging (CT with contrast or MRI with and without contrast) should be performed as clinically indicated. Chest imaging should be conducted every three to six months, including CT scans at least every six months for the initial five years, followed by yearly scans for a minimum of 10 years, based on clinical judgment. Assessment of function should be performed at each follow-up visit.
Recurrence	For locally recurrent tumors with resectable margins, wide excision with histologically negative surgical margins is recommended. If unresectable, consider radiation therapy. In cases of positive margins, consider radiation therapy or re-resection to achieve negative surgical margins. For cases with negative margins, observation may be appropriate. For systemic recurrence, follow the guidelines for metastatic chondrosarcoma management.

The partial removal of the sternum was necessary to ensure clear margins, which is critical to reduce the risk of disease recurrence. This presented a surgical challenge that required careful reconstruction to maintain stability and preserve lung function [5].

Studies have shown a correlation between the size of surgical margins and the likelihood of local recurrence, underscoring the importance of wide margins for better local control. The impact of surgical margins extends beyond managing disease, it also significantly affects overall survival. Our approach to the surgical management of sternal chondrosarcoma aligns with the recognized importance of achieving adequate margins. The partial removal of the sternum was not only aimed at ensuring clear margins but also at reducing the risk of recurrence in that specific area. Research indicates that patients who underwent surgery experienced a five-year survival rate improvement of 45%, supporting the effectiveness of this treatment strategy [10].

After the sternectomy procedure, it became necessary to reconstruct the chest wall due to the large bone defect resulting from the surgery. Defects larger than 5 cm may require reconstruction, and materials like polypropylene mesh and polytetrafluoroethylene have been successfully used for this purpose [7]. In our case, synthetic prostheses were used in line with the preference for polypropylene mesh due to its rigidity, inertness for tissue ingrowth, pliability for surgical shaping, and visibility on radiographs for effective monitoring. The polypropylene mesh acted as a scaffold for tissue growth and demonstrated effectiveness even in cases of infection, contributing to its preference in our reconstruction strategy [11].

After successful surgery and despite a favorable postoperative course, the patient experienced complications that required readmission to the hospital. Sternal wound infections are known to be severe complications that can occur after chest wall surgeries and carry a substantial risk of morbidity and mortality [12]. To address this complication, the patient was treated effectively with antibiotics, immediate wound revision, and partial omentoplasty. This strategy was not only aimed at resolving problems but also focused on preserving the synthetic prosthetic material. The challenges that arise when managing wound revisions in the context of chest wall surgeries emphasize the importance of immediate detection and intervention. This experience showcases how patient care is always changing and stresses the need for flexibility when dealing with evolving situations through postoperative care planning.

## Conclusions

This case highlights the intricate surgical management that contributed to the successful treatment of sternal chondrosarcoma, along with the effective management of postoperative complications. The successful treatment of the patient involved a comprehensive approach, emphasizing accurate diagnosis and surgical resection with thoughtful reconstruction, together with effective postoperative complication management. Sternal wound infection, a severe postoperative complication with a high mortality rate, requires prompt identification, precise revision with culture-directed antibiotics, and effort to preserve the prosthetic material. These findings provide insights into the most effective surgical strategies for treating sternal chondrosarcoma and highlight the significance of careful monitoring and individualized care to improve patient outcomes.
